# Advanced Nanobiomedical Approaches to Combat Coronavirus Disease of 2019

**DOI:** 10.1002/anbr.202000063

**Published:** 2021-01-18

**Authors:** Halle Lutz, Kristen D. Popowski, Phuong-Uyen C. Dinh, Ke Cheng

**Affiliations:** ^1^ Department of Molecular Biomedical Sciences North Carolina State University Raleigh NC 27607 USA; ^2^ Comparative Medicine Institute North Carolina State University Raleigh NC 27607 USA; ^3^ Joint Department of Biomedical Engineering University of North Carolina at Chapel Hill/North Carolina State University Raleigh/Chapel Hill NC 27607/27599 USA; ^4^ Division of Pharmacoengineering and Molecular Pharmaceutics University of North Carolina at Chapel Hill Chapel Hill NC 27599 USA

**Keywords:** coronavirus disease, exosomes, nanomedicine, severe acute respiratory syndrome coronavirus 2, therapeutics, vaccines

## Abstract

New infectious diseases are making themselves known as the human population grows, expands into new regions, and becomes more dense, increasing contact with each other and animal populations. Ease of travel has also increased infectious disease transmission and has now culminated into a global pandemic. The emergence of the novel coronavirus severe acute respiratory syndrome coronavirus 2 (SARS‐CoV‐2) in December 2019 has already infected over 83.7 million people and caused over 1.8 million deaths. While there have been vaccine candidates produced and supportive care implemented, the world is impatiently waiting for a commercially approved vaccine and treatment for the coronavirus disease of 2019 (COVID‐19). The different vaccine types investigated for the prevention of COVID‐19 all have great promise but face safety obstacles that must be first addressed. Some vaccine candidates of key interest are whole inactivated viruses, adeno‐associated viruses, virus‐like particles, and lipid nanoparticles. This review examines nanobiomedical techniques for combatting COVID‐19 in terms of vaccines and therapeutics.

## Graphical abstract

Advancements in nanotechnology offer multiple approaches for producing highly effective vaccines and biocompatible therapeutics to prevent, treat, and ultimately eradicate coronavirus disease of 2019 (COVID‐19). This review examines the benefits of promising vaccine candidates and the challenges each type of vaccine faces and provides an overview of innovative and safe methods for treating COVID‐19.

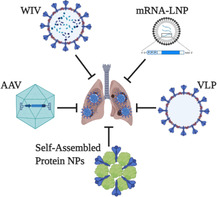

## 1 Introduction

Coronaviruses are single‐stranded positive‐sense RNA viruses ranging 80–120 nm in diameter.^[^ ^1^, ^2^, ^3^ ^]^ Severe acute respiratory syndrome coronavirus 1, or SARS‐CoV‐1, was the first highly virulent human coronavirus to appear in 2002 with a mortality rate of 9.6%.^[^ ^1^, ^3^, ^4^ ^]^ When Middle East respiratory syndrome coronavirus, or MERS‐CoV, produced an epidemic with a high mortality rate of 34.0%,^[^ ^5^ ^]^ being of the same coronavirus family, an inactivated SARS‐CoV‐1 vaccine was tested. However, it was found to have little effect on mice and ferrets, and it was determined that a new vaccine was required for protection from MERS‐CoV.^[^ ^6^ ^]^ In December 2019, the highly virulent novel severe acute respiratory syndrome coronavirus 2, SARS‐CoV‐2, was identified. Symptoms ranged among confirmed cases from asymptomatic to severe acute respiratory distress syndrome (ARDS), multiple organ failure, and even death.^[^ ^7^ ^]^ It quickly became clear that the elderly or those with contributed were more affected and contribute the most to the mortality rate, currently estimated to between 2% and 4%.^[^ ^8^ ^]^ In March 2020, the World Health Organization declared the coronavirus disease of 2019 (COVID‐19) a pandemic. Since then, an intense and thorough global research effort toward understanding this pathogen for vaccines and treatments has been conducted. So far, the preventative measures imposed have included requiring masks, encouraging physical distancing, and imposing lockdowns. While these measures have been effective in reducing the transfer of the virus and thereby reducing mortality due to COVID‐19, the global economy has been greatly impacted and producing a vaccine is critical. Early research discovered that SARS‐CoV‐2 used the same receptor as SARS‐CoV‐1 for viral entry, angiotensin‐converting enzyme 2 (ACE2). Decorating the surface of SARS‐CoV‐2 are spike proteins with two subunits, S1 and S2. On S1 is a receptor‐binding domain (RBD), which is the part of SARS‐CoV‐2 that binds to the ACE2 receptor for entry into host cells.^[^ ^9^ ^]^ This knowledge has provided researchers with a target for vaccination for the prevention of COVID‐19.

**Figure 1:**
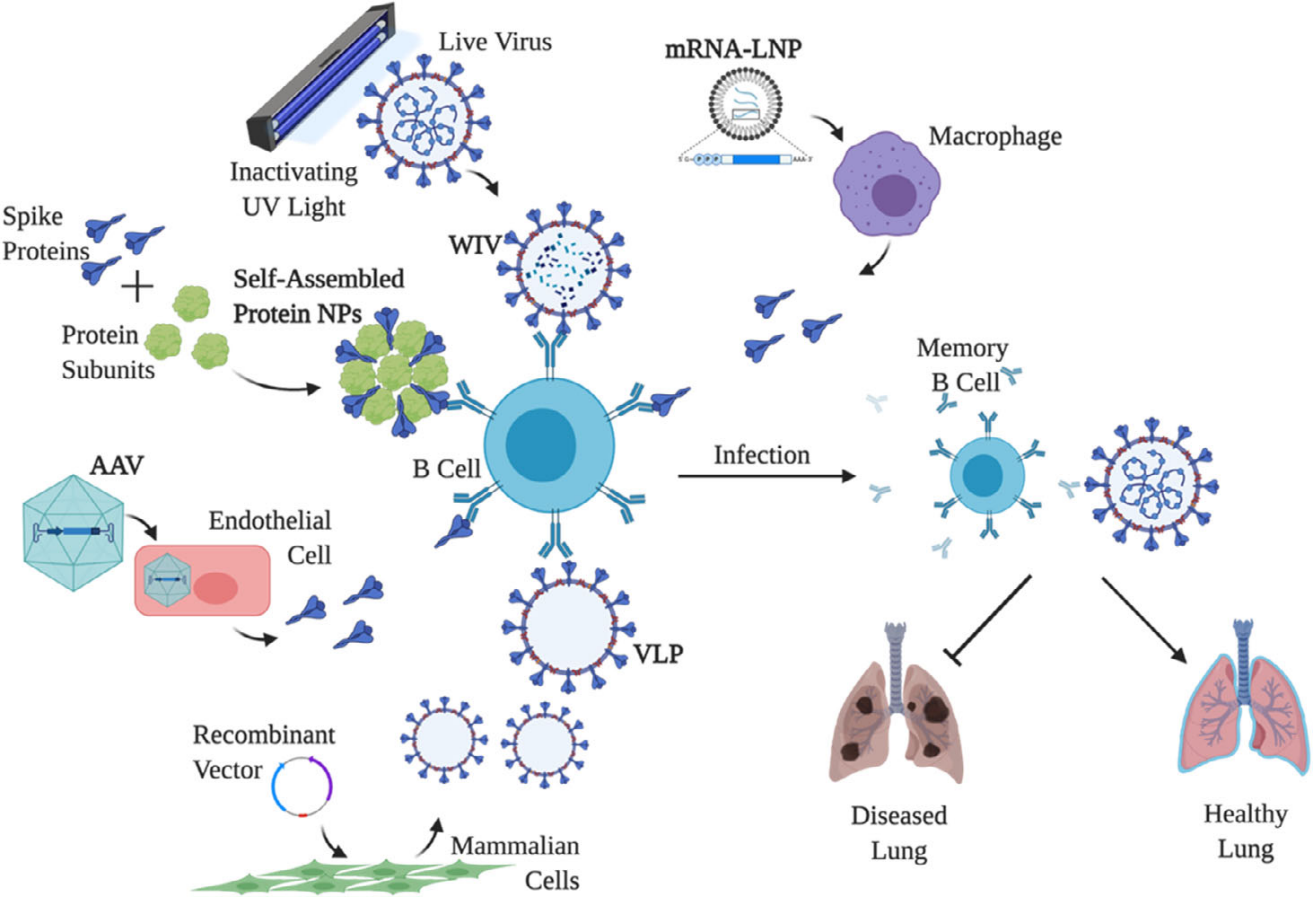
Nanobiomedical vaccine approaches for SARS‐CoV‐2. The vaccine mechanisms of WIVs, self‐assembled protein NPs, AAVs, VLPs, and mRNA‐LNPs for activating humoral and cellular immune responses.

Vaccination is understood to be one of the most effective methods for maintaining population health among both humans and livestock because infectious diseases are a major cause of death across all ages.^[^ ^10^ ^]^ Vaccines are a way of inducing the body to produce memory cells that make protective antibodies against infectious diseases. When the body does encounter the virus, the body can respond and eliminate the virus faster than it would have without a previous vaccination because the body already has memory cells to create the neutralizing antibodies. Vaccines are incredibly important as new infectious diseases emerge and threaten lives, standards of living, and the economy.^[^ ^7^, ^9^, ^10^ ^]^ Despite their necessity, vaccines are often difficult to produce in high enough quantity, and deliver at a reasonable cost.^[^ ^11^ ^]^ Moreover, conventional vaccines, such as whole inactivated virus (WIV) vaccines, typically have less efficacy due to inducing a weaker immune response. So far, only one human disease has successfully been eradicated, smallpox, and it was due to a global vaccination effort. A thorough understanding of the parent virus is critical and to obtain an adequate level of comprehension, BSL‐3 or BSL‐4 facilities to thoroughly study SARS‐CoV‐2.^[^ ^10^ ^]^ Vaccine development is critical to begin battling COVID‐19, but while vaccine candidates are tested, millions of people globally are suffering from COVID‐19. As of January 4, 2021, there have been over 83.7 million confirmed cases and over 1.8 million deaths from COVID‐19, and there is now only one approved treatment, Remdesivir.^[^ ^8^, ^12^ ^]^ So far, most treatments have been symptomatic, using mechanical ventilation or extracorporeal membrane oxygenation for patients, but it is clear that a COVID‐19‐specific treatment is needed. Nanobiomedical approaches to vaccine development—focusing on preventing viral entry or replication with self‐assembled protein nanoparticles (NPs) for example—and therapeutics are particularly promising because they are able to produce stronger responses with greater specificity for a longer period of time.^[^ ^13^, ^14^ ^]^ This review aims to describe the potential of nanobiomedical vaccine types and promising therapeutics for addressing SARS‐CoV‐2 (**Figure** **1**).

## 2 Nanobiomedical Approaches to COVID‐19 Vaccines

### 2.1 WIV for Infectious Diseases

Previously, vaccines for infectious diseases had primarily been live attenuated viruses, which posed a safety concern because the virus could reactivate and result in vaccine‐derived disease.^[^ ^6^, ^11^ ^]^ Inactivated vaccines were first administered in the late 1800s to protect against cholera, followed by diphtheria—both of which are bacterial pathogens. In the 1900s, inactivated vaccines were considered for viruses,^[^ ^15^ ^]^ and in 1936, the first WIV became available for the influenza virus, leading the way for other infectious diseases.^[^ ^16^, ^17^ ^]^ The main advantage of WIVs was that they worked much more safely while maintaining antigen presentation and stability for the immune system to respond.^[^ ^4^ ^]^ Another advantage is that with enough knowledge about the virus and replication process, large quantities of the virus can be quickly produced and therefore made quickly available.^[^ ^18^ ^]^ However, WIVs are not safe to produce and require culturing the infectious agents in at least BSL‐3 facilities, posing a risk to WIV developers.^[^ ^15^, ^19^, ^20^ ^]^ Frequently, mammalian cells, such as Vero, are used to culture the viruses.^[^ ^18^, ^20^ ^]^ To inactivate, live viruses are exposed to UV irradiation, formalin, beta‐propiolactone (BPL), or a combination of these to effectively damage the genetic material to prevent viral replication.^[^ ^18^ ^]^

To create a SARS‐CoV‐1 WIV, Spruth et al. utilized a double‐inactivation process of formalin and UV irradiation, having considered the virulence of SARS‐CoV‐1 and past reports of inactivation failures when only using one inactivation method. Despite the double inactivation, Spruth et al. successfully demonstrated that the WIV maintained its antigenic structure and immunogenicity.^[^ ^18^ ^]^ Unfortunately, in contrast, Bolles et al. demonstrated that double‐inactivated WIVs have the potential to actually cause a hypersensitive response, emphasizing the importance of careful vaccine design.^[^ ^21^ ^]^ To overcome this, an adjuvant is recommended, which will stimulate a T‐cell response.^[^ ^22^ ^]^ WIVs have been criticized for their poor immunogenicity and the subsequent necessary booster immunizations, but they have consistently been shown to produce neutralizing antibodies.^[^ ^10^, ^19^ ^]^ When studying a BPL‐inactivated SARS‐CoV‐1 WIV in hamsters, Roberts et al. found that even low levels of neutralizing antibodies conferred protection.^[^ ^4^ ^]^ In further support of WIV efficacy, the poliovirus epidemic in the United States was significantly attenuated by an inactivated polio vaccine. Before another vaccine candidate had been approved, the polio WIV was responsible for reducing paralytic polio by about 94% from 13.9 cases per 100 000 people to a mere 0.8 cases per 100 000 people.^[^ ^15^ ^]^ Since the poliovirus epidemic, the only other WIVs to become commercially available are for Hepatitis A,^[^ ^23^ ^]^ rabies,^[^ ^24^ ^]^ and influenza virus.^[^ ^25^ ^]^ Still, WIVs continue to be studied as potential vaccine candidates for old and emerging viral diseases, including the SARS and MERS coronaviruses. Until the viruses are completely eliminated, different vaccination techniques to combat these viruses will continue to be investigated, but for the COVID‐19 pandemic, SARS‐CoV‐2 WIVs may be a temporary solution. Already, Gao et al. have shown that after three SARS‐CoV‐2 WIV immunizations, rhesus macaques demonstrated complete protection when challenged with SARS‐CoV‐2.^[^ ^26^ ^]^ In fact, Sinovac's CoronaVac is a WIV for immunization against SARS‐CoV‐2 and has entered a phase III clinical trials, having previously shown safety and efficacy in a randomized, double‐blind phase I/II clinical trial.^[^ ^27^ ^]^

### 2.2 Adeno‐Associated Virus for Infectious Diseases

In 1965, adeno‐associated virus, or AAV, was discovered in a sample of adenovirus, and characterized as a replication‐defective *Parvoviridae* virus that was nonenveloped and contained either positive‐ or negative‐sense single‐stranded DNA.^[^ ^28^, ^29^, ^30^ ^]^ The genome contains genes for replication, the capsid proteins, and assembly with inverted terminal repeats at the beginning and end.^[^ ^28^, ^29^, ^30^, ^31^ ^]^ AAV is a safe technology because it lacks the ability to replicate without the genes of a helper virus, such as an adenovirus, and even in the presence of an adenovirus, AAV has not been associated with any disease.^[^ ^31^, ^32^ ^]^ In fact, Naso et al. note that most people have already been exposed to an AAV variant but no noticeable symptoms have resulted.^[^ ^30^ ^]^ Therefore, it has been mainly used as a delivery system for either gene therapy or antigens for immunogenicity.^[^ ^28^, ^29^, ^30^, ^31^, ^32^, ^33^, ^34^ ^]^ Gene therapy has focused on cancer and neurodegenerative diseases, using recombinant AAV, in which the viral genetic code is replaced with a gene of interest.^[^ ^31^, ^35^ ^]^ However, the replacement gene must be similar or smaller than the 4.7 kb AAV genes or the stability of the recombinant AAV will suffer.^[^ ^28^, ^29^, ^30^, ^31^ ^]^ Furthermore, AAV production is difficult to scale up because even though mammalian and insect cells can produce high quantities in a short amount of time, the AAV replication gene kills the cells, making it an expensive therapy.^[^ ^30^ ^]^ So far, only two AAVs have been commercially approved, but each cost approximately 1 million dollars for a single treatment.^[^ ^28^, ^29^, ^30^, ^31^ ^]^ Still, AAV has been shown to enable targeted delivery and expression to specific tissues as a result of the AAV variant combined with the tissue‐specific promoter sequence, and over 200 clinical trials using AAVs are in progress.^[^ ^30^ ^]^

AAV has been criticized as a vaccine option due to its naturally poor immunogenicity that is furthered by preexisting immunity due to a previous exposure. Furthermore, Lin et al. explain that at least two AAV immunizations must occur for a detectable antibody response in human immunodeficiency virus 1 (HIV‐1), and considering the current AAV therapy costs, it is not likely feasible.^[^ ^28^ ^]^ However, antigens bound to the surface or the gene an AAV carries can result in antigen‐presenting cell (APC) uptake and recruit B and T cells for a strong immune response, and different expression systems are still being explored.^[^ ^30^ ^]^ In terms of immunogenicity, an AAV carrying a gene coding for a neutralizing antibody can be used to protect against pathogenic infections.^[^ ^31^ ^]^ In fact, Demminger et al. used a recombinant AAV vector carrying an influenza hemagglutinin expression cassette to show that broadly reactive neutralizing antibodies were induced and protected against influenza viruses with different mutations.^[^ ^34^ ^]^ In addition, Yusuf et al. have demonstrated that recombinant AAV‐based immunization after adenovirus priming was able to induce a long‐lasting antibody response in a mouse model.^[^ ^32^ ^]^ Recombinant AAV technology has been considered for SARS‐CoV‐1 with promising results. Du et al. found that intranasal vaccination of a recombinant AAV encoding the receptor‐binding domain of the SARS‐CoV‐1 spike protein induced a strong systemic humoral response that was comparable to intramuscular vaccination. Unfortunately, the intranasal administration resulted in a shorter immune response than intramuscular vaccination, indicating that multiple immunizations would be necessary, again making production costs a consideration.^[^ ^33^ ^]^ Considering the scale of the COVID‐19 pandemic, for AAV vaccination to become a truly viable option as a COVID‐19 therapeutic, an inexpensive yet high‐quality production method must be identified. Two noteworthy COVID‐19 AAVs are those created by the Gamaleya Research Institute of Epidemiology and Microbiology and AstraZeneca, both of which seek to immunize against the SARS‐CoV‐2 spike protein.^[^ ^36^, ^37^ ^]^ AstraZeneca has entered a phase III clinical trial, but not yet reported efficacy, while the Gamaleya Research Institute of Epidemiology and Microbiology reported that their adenoviral‐based vaccine, Gam‐COVID‐Vac or Sputnik V, has 92% efficacy from 20 patients in their phase III clinical trial.^[^ ^38^, ^39^ ^]^

### 2.3 Virus‐Like Particles for Infectious Diseases

Virus‐like particles, or VLPs, were first described in 1948 when studying adenocarcinoma,^[^ ^40^ ^]^ and in 2006, VLPs gained recognition when it was commercially approved as a synthetic vaccine to protect against the human papillomavirus (HPV).^[^ ^41^ ^]^ Despite no longer being a new technology, VLPs have recently seen a rapid expansion for the treatment and prevention of both noninfectious and infectious diseases for humans and livestock. VLPs are typically 20–100 nm, mimicking the size of the parent virus and allowing VLPs to enter the lymph nodes to induce B‐cell activation, APC uptake, and subsequently, T‐cell activation.^[^ ^42^, ^43^ ^]^ VLPs are composed of one or more repeating viral capsid and structural proteins,^[^ ^11^ ^]^ meaning that different VLPs can be created from a single virus if different capsid proteins or different combinations of capsid proteins are used.^[^ ^44^, ^45^ ^]^ For instance, HPV has two capsid proteins, L1 and L2. Current commercially available HPV vaccines are VLP‐based and built with the L1 capsid antigen, leaving the possibility of an L2‐based VLP to overcome the type‐restriction protection the L1‐based VLPs face.^[^ ^45^ ^]^ Because VLPs carry the same antigens as the parent virus, they induce the same immunogenicity.^[^ ^46^ ^]^ In fact, because the antigen is repeated so many times in such high density, VLPs can actually amplify their immunogenicity in comparison with the parent virus.^[^ ^45^ ^]^ VLP activation of the immune system is strong enough to provide protection for years, with the first VLP‐based vaccine for the Hepatitis E virus conferring protection for almost 5 years after the initial vaccination and VLP‐based vaccines for HPV offering protection for up to a decade.^[^ ^11^, ^45^ ^]^ The chief difference from the parent virus is that VLPs lack the genetic core that allows viruses to replicate, making VLPs a safer vaccine alternative than current options.^[^ ^47^, ^48^ ^]^ However, VLPs are not just being studied because they are safe and effective compared with virus‐related vaccines. There have also been investigations into how VLPs can be used as NPs for targeted delivery of drugs,^[^ ^44^ ^]^ gene therapy,^[^ ^47^ ^]^ and the treatment of noninfectious diseases such as cancer,^[^ ^43^, ^45^, ^48^ ^]^ neurodegenerative diseases,^[^ ^49^, ^50^ ^]^ hypertension,^[^ ^51^, ^52^, ^53^ ^]^ nicotine addiction,^[^ ^54^ ^]^ diabetes,^[^ ^55^ ^]^ and even allergies.^[^ ^50^, ^56^ ^]^

VLPs are relatively inexpensive to produce, but the production process itself is complicated.^[^ ^47^ ^]^ VLPs self‐assemble and can range from a simple single‐layered VLP to a complex multilayered VLP composed of multiple proteins or decorated with various glycoproteins, depending on the parent virus and the purpose of the VLP. Once thorough investigation into the parent virus is concluded, only BSL‐2 facilities are necessary for VLP construction. The size and geometry of VLPs are important for B‐cell activation and long‐lasting antibody responses.^[^ ^42^ ^]^ The addition of peptides to carrier coat proteins cannot interfere with the ability to self‐assemble.^[^ ^47^ ^]^ The recombinant proteins that self‐assemble into VLPs can be expressed in several host systems, including bacteria, yeast, insects, plants, mammalian cells, and even cell‐free systems. ^[^ ^10^, ^11^, ^47^, ^57^, ^58^ ^]^ In addition, the same VLP can be produced in different host systems.^[^ ^45^ ^]^

VLPs can be modified to further increase immunogenicity by adding antigens or epitopes with genetic fusions or chemically conjugating to preformed VLPs.^[^ ^10^, ^11^, ^44^ ^]^ Furthermore, VLPs can be modified to have an increased diversity of antigens.^[^ ^42^ ^]^ Other modifications are necessary for increasing the stability of VLPs in terms of temperature, pH, and time.^[^ ^43^, ^44^, ^47^ ^]^ However, the modifications made must be done with careful consideration because VLPs’ ability to self‐assemble and stability depends on the conformation of the VLP. Introducing too many components has the potential to render the VLP unable to assemble and thus ineffective.^[^ ^42^ ^]^ In fact, Boigard et al. found that the temperature at which the VLP is produced can also impair VLP efficacy. When studying dengue VLPs in a mouse model, only VLPs produced at 31 °C, as opposed to 37 °C, were able to generate neutralizing antibodies.^[^ ^59^ ^]^ Computational models that study protein folding are recommended to save money and time and ultimately increase the success rate of VLPs becoming candidates for clinical trials.^[^ ^10^ ^]^ Despite the difficulties of producing VLPs, VLPs are still considered easier to produce than their native virus.

There are multiple procedures after producing a VLP to ensure its purity and safety, as well as to ensure its function. Some procedures used for VLP purification are chromatography,^[^ ^44^, ^47^, ^57^ ^]^ sucrose density gradients,^[^ ^1^, ^6^, ^57^ ^]^ and centrifugation. To confirm purity, sodium dodecyl sulfate polyacrylamide gel electrophoresis is frequently used.^[^ ^10^, ^11^, ^53^ ^]^ Steps required for VLP characterization are as specific as identifying the primary amino acid sequence,^[^ ^11^ ^]^ molecular weight by mass spectrometry^[^ ^50^ ^]^ and Western blots,^[^ ^1^, ^6^ ^]^ and determining the isoelectric point.^[^ ^11^, ^47^ ^]^ To confirm VLP structure, transmission electron microscopy and cryoelectron microscopy are typically used.^[^ ^1^, ^10^, ^47^ ^]^ Immunogenicity testing is assessed with enzyme‐linked immunosorbent assays (ELISAs),^[^ ^1^, ^6^, ^11^, ^47^, ^57^ ^]^ probing with monoclonal antibodies,^[^ ^11^ ^]^ and immunodiffusion tests,^[^ ^10^ ^]^ before an appropriate preclinical animal model is used to accurately predict the safety and efficacy of the VLPs.^[^ ^6^, ^10^ ^]^

Some of the viruses that VLPs have been used for include HPV, influenza viruses, flaviviruses, and coronaviruses. HPV has been of particular interest because these double‐stranded DNA viruses are the primary cause of cervical cancer, which is the third deadliest cancer for women. As mentioned earlier, the currently approved HPV vaccines were constructed from only one of the two HPV capsid proteins. Therefore, even though they are effective for up to 10 years, the use of a single capsid protein prevents the vaccines from offering general protection against all types of HPV.^[^ ^45^ ^]^ The influenza viruses present another enduring viral challenge because hemagglutinin, the glycoprotein responsible for entry, rapidly mutates.^[^ ^60^ ^]^ As a result, new influenza vaccines must be made each year to maintain protection from the new changes in hemagglutinin conformation. Even then, vaccine production lags behind influenza virus changes.^[^ ^61^ ^]^ It has been suggested that using multistructural VLPs as vaccines, instead of the classic influenza vaccine method using embryonated chicken eggs, could produce immune responses that would defend against the different forms of influenza.^[^ ^34^, ^60^, ^62^, ^63^ ^]^ More investigation is necessary, but influenza VLPs have the potential to save time and labor spent continuously developing subtype‐ and strain‐specific influenza vaccines. Even as VLPs are used to address challenges that plagued for centuries, they are also being considered as novel infectious diseases emerge. Flaviviruses have grown as a global health concern due to the expanding human population into tropical and subtropical regions, where mosquitos carry and transmit flaviviruses.^[^ ^64^, ^65^ ^]^ Flaviviruses are single‐stranded positive‐sense RNA viruses that include dengue virus, West Nile virus, and Zika virus.^[^ ^26^, ^57^, ^64^, ^65^ ^]^ Flavivirus VLPs have been demonstrated to produce strong, long‐lasting immunogenicity and many have been brought to clinical trials, with Zika virus and chikungunya VLP vaccines currently in phase 2.^[^ ^66^ ^]^ Among emerging infectious diseases are coronaviruses, and in December 2019, coronaviruses were brought to world attention again as the third highly infectious and deadly novel coronavirus in two decades appeared.

Since the SARS‐CoV‐1 outbreak, many vaccine candidates have been created with the expectation of another outbreak, one of which was a VLP produced by Lu et al., which was composed of the spike, membrane, and envelope proteins. Lu et al. tested the immunogenicity of their VLP and found that only four subcutaneous immunizations over the course of a month produced high immunogenicity with increased interferon (IFN)‐gamma production and T helper type 1 (Th1) cell activity. In fact, the antibodies produced were able to neutralize up to 90% of the SARS pseudotypes.^[^ ^1^ ^]^ With the advent of MERS‐CoV, Wang et al. produced a VLP similar to the aforementioned SARS‐CoV VLP consisting of spike, envelope, and membrane proteins. When nonhuman primates were immunized with the MERS‐CoV VLP, they found that immunization successfully produced high titers up to 8 weeks after immunization and that IFN‐gamma production was increased.^[^ ^6^ ^]^ Further exploring the application of VLPs for coronaviruses, Chen et al. created and studied a VLP decorated with avian infectious bronchitis virus (IBV). Covering gold NPs with IBV spike protein, the VLP effectively delivered the antigens to the lymph nodes and resulted in increased IFN‐gamma levels. Clinical scores were also reduced in the chickens that received the IBV‐VLP as opposed to free protein or the inactivated virus.^[^ ^67^ ^]^ Therefore, VLPs have great potential to become an effective COVID‐19 vaccine.

### 2.4 Lipid NPs for mRNA Vaccine Delivery

Lipid NPs, or LNPs, carrying mRNA recently emerged in the 1990s as safe, efficient, easy to manufacture, and scalable vaccination options.^[^ ^9^, ^68^ ^]^ Since then, an mRNA‐encapsulated LNP (mRNA‐LNP) has not yet been made commercially available, but an interference RNA‐encapsulated LNP (RNAi‐LNP), Onpattro, was recently approved for polyneuropathy.^[^ ^69^ ^]^ mRNA can be either nonreplicating or self‐replicating, with the classification primarily depending on whether the nucleosides were modified.^[^ ^68^, ^70^ ^]^ As for the LNPs, they can be composed of a lipid monolayer or a bilayer. mRNA‐LNPs are administered by injection, either intramuscular or intradermal, and travel to enter the lymph nodes where mRNA‐LNPs are then taken up by APCs. Once the mRNA enters the cytoplasm, the host cell's machinery is used to translate the corresponding protein and the protein expression activates B and T cells.^[^ ^9^, ^68^ ^]^ mRNA‐LNPs have been so effective in causing potent immunogenicity and long‐lasting neutralizing antibody protection that only one or two doses have been needed to protect against infectious diseases in flavivirus,^[^ ^71^ ^]^ HIV‐1,^[^ ^72^ ^]^ influenza,^[^ ^63^ ^]^ and SARS‐CoV‐2^[^ ^2^, ^9^, ^70^ ^]^ animal models. One of the reasons mRNA‐LNPs have such high efficacy is the stability of the mRNA due to structural modifications and encapsulation within a NP that protect it from degradation.^[^ ^9^ ^]^ Moreover, mRNA‐LNPs are flexible: for instance, a Zika virus mRNA platform was adapted for a different flavivirus, Powassan virus, and was able to produce a strongly immunogenic response.^[^ ^73^ ^]^ Still, mRNA‐LNPs are not without drawbacks, one of which involves production of antibodies that protect against one disease but may also increase susceptibility to another disease. This effect was observed with Zika virus mRNA‐LNPs that increased dengue virus infection.^[^ ^71^ ^]^

The high production rate of mRNA‐LNPs is particularly attractive when considering the global impact of the SARS‐CoV‐2 pandemic because it means an mRNA‐LNP vaccine would be both affordable and easily distributed. Currently, mRNA‐LNPs are considered the forerunner for a SARS‐CoV‐2 vaccine, as Moderna has entered a phase III clinical trial with an mRNA‐LNP vaccine encoding the spike protein, mRNA‐1273.^[^ ^2^, ^9^ ^]^ Many other research groups have also produced their own SARS‐CoV‐2 mRNA‐LNP, exploring different targets from just the receptor‐binding domain to the entire spike protein, such as BioNTech/Pfizer's BNT162b2, which has also entered a phase III clinical trial.^[^ ^74^ ^]^ Some are considering other characteristics of a SARS‐CoV‐2 vaccine that would be important in addition to producing a high titer of neutralizing antibodies such as thermostability. Zhang et al. not only created a SARS‐CoV‐2 mRNA‐LNP that demonstrated complete protection in a mouse model, but also created a SARS‐CoV‐2 mRNA‐LNP that was stable when stored at room temperature for a week (**Table** **1**).^[^ ^2^ ^]^

**Table 1:** Applications of different vaccine types. Examples of preclinical and clinical applications for which WIVs, AAVs, VLPs, and mRNA‐LNPs have been used

Vaccination method	Disease	Stage	Citation
WIVs	Poliovirus	FDA approved	Karch et al. (2016)^[^ ^15^ ^]^
	Hepatitis A	FDA approved	Herk et al. (2005)^[^ ^23^ ^]^
	Rabies	FDA approved	States et al. (1998)^[^ ^24^ ^]^
	Influenza	FDA approved	Statler et al. (2019)^[^ ^25^ ^]^
	MERS‐CoV	Preclinical	Deng et al. (2018)^[^ ^22^ ^]^
	SARS‐CoV‐1	Preclinical	Spruth et al. (2006)^[^ ^18^ ^]^
	SARS‐CoV‐2	Phase III	Zhang et al. (2020)^[^ ^27^ ^]^
AAVs	Lipoprotein lipase deficiency	EMA approved	Santiago‐Ortiza et al. (2016)^[^ ^31^ ^]^
	Leber congenital amaurosis	FDA approved	Wang et al. (2019)^[^ ^29^ ^]^
	HIV‐1	Preclinical	Lin et al. (2018)^[^ ^28^ ^]^
	Influenza	Preclinical	Demminger et al. (2020)^[^ ^34^ ^]^
	SARS‐CoV‐1	Preclinical	Du et al. (2008)^[^ ^33^ ^]^
	SARS‐CoV‐2	Phase III	Logunev et al. (2020)^[^ ^36^ ^]^
			Folegatti et al. (2020)^[^ ^37^ ^]^
VLPs	HPV	FDA approved	Wang et al. (2013)^[^ ^45^ ^]^
	Flaviviruses	Phase I	Ramanathan et al. (2018)^[^ ^66^ ^]^
	Influenza	Preclinical	McCraw et al. (2018)^[^ ^52^ ^]^
			Buffin et al. (2019)^[^ ^54^ ^]^
	MERS‐CoV‐1	Preclinical	Wang et al. (2017)^[^ ^6^ ^]^
	SARS‐CoV‐1	Preclinical	Lu et al. (2007)^[^ ^1^ ^]^
mRNA‐LNPs	HIV‐1	Preclinical	Pardi et al. (2019)^[^ ^72^ ^]^
	Flaviviruses	Preclinical	Richner et al. (2017)^[^ ^71^ ^]^
	Influenza	Phase I	Bahl et al. (2017)^[^ ^63^ ^]^
	SARS‐CoV‐2	Phase III	Zhang et al. (2020)^[^ ^2^ ^]^
			Wang et al. (2020)^[^ ^9^ ^]^
			Walsh et al. (2020)^[^ ^74^ ^]^

## 3 Nanobiomedical Approaches to COVID‐19 Therapeutics

### 3.1 Exosomes as a Therapeutic and Drug Delivery Platform

Exosomes are a type of extracellular vesicle typically 30–100 nm in diameter that are released by cells.^[^ ^75^ ^]^ Originally thought to be carrying cell waste, exosomes were not considered for any application until recently when they were discovered to carry DNA, RNA, and proteins for cell–cell signaling.^[^ ^76^ ^]^ Natural exosomes have demonstrated their regenerative potential in a variety of diseases,^[^ ^77^ ^]^ but especially in lung diseases, both acute and progressive, such as ARDS, chronic obstructive pulmonary disease, and idiopathic pulmonary fibrosis.^[^ ^5^, ^78^, ^79^ ^]^ In fact, the beneficial effects of mesenchymal stem cell (MSC) transplants have been largely attributed to their exosomes.^[^ ^5^, ^75^ ^]^ Furthermore, unlike MSCs, exosomes are very stable, and, though still difficult and time‐consuming, easier to scale up to necessary dosage with bioreactors.^[^ ^6^, ^7^, ^65^ ^]^ Exosomes are also produced by all cells and are present in almost all biofluids.^[^ ^12^, ^75^ ^]^ Though there are concerns that exosomes package, deliver, and, thus, amplify viral load throughout the body, the low immunogenicity of exosomes means that using exosomes from a healthy, uninfected source to combat COVID‐19 should be safe and not cause adverse events.^[^ ^12^ ^]^ In May 2020, a phase I clinical trial was completed, successfully demonstrating the safety and efficacy of bone marrow MSC exosomes for treating COVID‐19, in which all patients responded positively with clinical improvement.^[^ ^80^ ^]^ In July 2020, a group from Ruijin Hospital, China, completed a phase I clinical trial to determine the safety and efficacy of adipose MSC exosomes in treating COVID‐19‐associated pneumonia.^[^ ^81^ ^]^ This same group has also begun a phase I clinical trial focusing on applying MSC exosomes as a treatment for ARDS, another lung disease closely tied to COVID‐19.^[^ ^82^ ^]^ As exosomes have parent cell‐specific functions based on their cargo and membrane proteins, our group developed lung spheroid cells (LSCs) to study their effect in lung disease models.^[^ ^83^, ^84^ ^]^ We found that not only are LSCs more effective than MSCs at attenuating bleomycin‐induced pulmonary fibrosis, but also that the LSC exosomes attenuated bleomycin and silicosis pulmonary fibrosis in mouse models.^[^ ^79^ ^]^ Studying the therapeutic effect of LSC exosomes in COVID‐19 and comparing it with bone marrow MSC exosome results are the next logical steps.

Exosomes can also be engineered to modify their cargo or the proteins in their lipid membrane, therefore enabling exosome functions to be altered. For instance, exosomes have been modified to display the tumor necrosis factor‐alpha receptor 1 as a way of reducing inflammation.^[^ ^85^, ^86^, ^87^ ^]^ As a result, many researchers are currently investigating how exosomes can be used as viral decoys. Viral decoys have been previously used for HIV‐1 with T‐cell membrane‐coated NPs,^[^ ^88^ ^]^ for adenovirus‐37 with sulfated glycosaminoglycans,^[^ ^89^ ^]^ and for A disintegrin and metalloprotease domain‐containing protein 10 (ADAM10) expressing exosomes with methicillin‐resistant staphylococcus aureus.^[^ ^90^ ^]^ Recently, nanodecoys or decoy cells have been considered for SARS‐CoV‐2 by engineering cells that do not replicate and present immunogenic antigens.^[^ ^91^, ^92^ ^]^ However, exosomes are the most logical choice for acting as a viral decoy because their membrane proteins already reflect those in their parent cells, which can include the ACE2 receptor, which is what SARS‐CoV‐2 uses for viral entry.^[^ ^2^, ^5^, ^7^, ^9^, ^12^, ^80^ ^]^ In May 2020, the idea of modifying exosome membranes to increase ACE2 and using the receptor to trick the virus into entering exosomes instead of cells was proposed.^[^ ^93^ ^]^ This could be a very effective method because using lung cell‐derived exosomes would increase targeting to the lung. Furthermore, if exosome cargo was also engineered to include an antiviral drug to destroy the virus once trapped, the virus would not be able to infect host cells if exosomes released them.

Current NPs used for drug delivery have problems with biocompatibility, structural heterogeneity, maintaining stability and solubility for delivery, avoiding clearance, reaching target regions, and result in low specificity and high toxicity.^[^ ^76^ ^]^ Frequently, NPs for delivering chemotherapeutics depend on the enhanced permeability and retention (EPR) effect, but this is a passive and slow process. Exosomes have demonstrated biocompatibility and low immunogenicity, and exosomes are able to cross the blood‐brain barrier.^[^ ^78^ ^]^ More importantly, because exosomes are the body's mechanism for cell signaling, exosomes naturally target specific tissues. Therefore, exosomes have been investigated for targeted drug delivery, especially since it was found that using exosomes to deliver paclitaxel for treating multiple drug resistance cancer enhanced paclitaxel potency.^[^ ^94^ ^]^ Exosomes that carry and deliver antiviral drugs to COVID‐19 or other virus‐infected patients have potential to be a safe, strong‐acting therapeutic.

### 3.2 LNPs as a Drug Delivery Platform

When considering antiviral drugs, a delivery platform is required because antivirals face many challenges once administered including crossing the blood–brain barrier and maintaining stability and efficacy upon dilution. One solution is in lipid NPs, which are biocompatible and protect the drug during transport.^[^ ^95^, ^96^ ^]^ LNPs are especially useful for drug delivery because their nanoscale size enables access into cells to release the unaltered cargo.^[^ ^97^ ^]^ Unfortunately, an important consideration with LNPs is the rate at which the drugs are released, with Naahidi et al. noting that sometimes LNPs may release the drug at a rate that is too low to produce an effect.^[^ ^98^ ^]^ Previously, we described using LNPs to deliver mRNA that would produce proteins that induce an immune reaction against SARS‐CoV‐2, but LNPs can also package mRNA as a COVID‐19 treatment, as well as antivirals and proteins to combat COVID‐19. For instance, Kim et al. used mRNA‐LNPs to induce cells to produce soluble human ACE2, which bound to SARS‐CoV‐2 and inhibited SARS‐CoV‐2 pseudovirus infection.^[^ ^99^ ^]^ LNPs have also been noted for carrying antiviral drugs, such as azidothymidine (AZT) and (S)‐1‐(3‐hydroxy‐2‐phsophonylmethoxypropyl)cytosine (HPMPC), to inhibit viral replication in HIV and herpes simplex virus (HSV), respectively.^[^ ^100^, ^101^ ^]^ Some FDA‐approved treatments using LNPs to package and deliver drugs include Doxil Caelyx and Myocet—or LNP encapsulated doxorubicin—for various cancers, Marqibo—or LNP encapsulated vincristine—for various cancers, and Mepact—or LNP encapsulated mifamurtide—for osteosarcomas.^[^ ^102^, ^103^ ^]^ Moreover, as an antiviral for flavivirus dengue virus, Croci et al. used LNPs to encapsulate and reduce the cytotoxicity of ivermectin, which has recently been proposed for COVID‐19 treatment due to its efficacy against both RNA and DNA viruses.^[^ ^104^, ^105^ ^]^ Another application of LNPs is as a drug carrier for Remdesivir, which has shown efficacy against COVID‐19 in vitro and in COVID‐19 patients in clinical trials.^[^ ^106^, ^107^ ^]^ Therefore, LNPs offer a safe and effective way to deliver and preserve the potency of COVID‐19 drugs.

## 4 Conclusion

Since the advent of SAR‐CoV‐2, researchers have come together in a global effort and tirelessly pursued vaccine and therapeutic options, considering repurposing current drugs or creating new ones. According to clinicaltrials.gov, there are 266 vaccine studies with 62 currently in phase III, meaning that these vaccines have demonstrated both safety and efficacy and are now being tested for comparison against placebo vaccinations.^[^ ^108^ ^]^ Candidates from each type of vaccine described in this review have reached phase III, illustrating the potential for each and the potential for a SARS‐CoV‐2 vaccine to become available soon. In fact, if Moderna's vaccine passes phase III, the US Department of Health and Human Services has estimated that by April there will be enough doses to treat all Americans.^[^ ^109^ ^]^ As for therapeutics, the innate regenerative properties and ability to carry drugs to targeted locations without causing immunogenicity or other undesirable side effects make exosomes a prime therapeutic.^[^ ^75^, ^76^ ^]^ Therefore, we believe exosomes hold the potential as a nanomedicine drug carrier to deliver COVID‐19 therapeutics.^[^ ^110^, ^111^ ^]^

## Conflict of Interest

The authors declare no conflict of interest.
